# The Therapeutics of Tetanus

**Published:** 1919

**Authors:** 


**The Therapeutics of Tetanus**. By HERMANN B. GESSNER. *Journal of the American Medical Association*, Sept. 14, 1918.

We must admit that we have progressed but a little in the treatment of this disease, and that strenuous efforts must be made to correct our errors and lessen the heavy mortality rate.

Experience has shown that in patients presenting likelihood of tetanus infection the preventive dose should be repeated after ten days, if suppuration continues. Its prompt administration, even after the lapse of several days, may avert the disease or diminish its severity.

All victims of accidental injury of a punctured, lacerated, crushed or gun-shot character, especially when associated with foreign bodies, or with exposure to street, garden or stable contamination, should receive 1500 units of anti-tetanic serum at first treatment.

It may be well to refer to the question of anaphylaxis and primary hypersensitiveness to serum. The author has never known a death to result from either cause. In all patients exposed to tetanus infection the serum should be given regardless of the remote danger from animal proteins.

If suppuration continues, the administration of the serum should be repeated at intervals of ten days, as we have reason to believe that its protective influence does not last beyond this time.

Treatment should be by large doses of serum of not less than 10,000 units to the dose. Administration by the intravenous, intraneural, intramuscular and subarachnoid methods should be more extensively employed for the purpose of bringing out their value more thoroughly.

Another element in the treatment of tetanus, which does not appear from these figures, lies in the personal equation of the attending surgeon and nurses. These patients require detailed care for nutrition, skin cleansing, bowels, etc., which is very laborious. They need the greatest degree of quietness and gentleness. Too often their cases are looked on as necessarily fatal, and, as unwelcome guests, they are screened off not only from view but also from active attention. Sedatives should be used to calm anxiety and relieve pain. These cases should be concentrated in the hands of attendants sanguine of obtaining good results by hard work, and anxious to lessen the mortality of this dread disease.

